# Urinary cortisol and depression in early pregnancy: role of adiposity and race

**DOI:** 10.1186/s12884-015-0466-7

**Published:** 2015-02-13

**Authors:** John W Luiza, Marcia J Gallaher, Robert W Powers

**Affiliations:** Department of Obstetrics, Gynecology and Reproductive Sciences, Pittsburgh, Pennsylvania USA; Magee-Womens Research Institute, University of Pittsburgh School of Medicine, Pittsburgh, Pennsylvania USA

**Keywords:** Pregnancy, Depression, Obesity, Cortisol, Race

## Abstract

**Background:**

Depression before and during pregnancy is associated with adverse birth outcomes including low birth weight and preterm birth. Abnormal maternal cortisol has been hypothesized as one mediator between depression and adverse birth outcomes. The relationship between cortisol and depression in pregnancy is exhibited most strongly in the African American population, and most studies have focused either on circulating or placental levels of cortisol. The utility of urinary cortisol in early pregnancy related to depression and adiposity has not been investigated.

**Methods:**

Twenty-five pregnant African American women identified by the Edinburgh Depression Scale as having depression were investigated and matched by body mass index (BMI), age, race, and infant birth weight centile to non-depressed subjects. Maternal urine and plasma cortisol in early pregnancy were quantified and investigated in relation to depression and adiposity.

**Results:**

Morning urine cortisol levels tracked positively with plasma cortisol (r^2^ = 0.25, p < 0.001). However, no differences were observed in either urinary or plasma cortisol between depressed and non-depressed pregnant women. Plasma cortisol was significantly negatively associated with several measures of maternal adiposity including percent body fat (r^2^ = −0.10, p < 0.05), however this relationship was present only in the non-depressed women. In a post-hoc analysis, non-depressed non-obese women were found to have significantly higher cortisol levels compared to women with depression, obesity or both (p < 0.05).

**Conclusions:**

Depressed pregnant women and non-depressed obese pregnant women evidence atypical cortisol levels compared to non-depressed non-obese pregnant women. Plasma cortisol in early pregnancy is negatively associated with measures of maternal adiposity. Atypical low circulating maternal cortisol among depressed (lean and obese) and non-depressed obese pregnant African American women may indicate hypothalamic-pituitary axis dysfunction in early pregnancy.

## Background

Postnatal depression is an important focus of appropriate clinical care for expectant mothers; however, antenatal depression is also of clinical concern [1]. Depression during pregnancy has been linked to pregnancy complications including preeclampsia, and adverse birth outcomes such as preterm birth and low birth weight which are leading causes of pregnancy related morbidity and mortality [2]. The link between maternal depression and adverse birth outcomes is unclear, and several factors have been examined including inflammation and dysregulation of the hypothalamic-pituitary axis (HPA) [3,4]. Depression and anxiety during pregnancy have been associated with elevated maternal cortisol levels and a negative evaluation of pregnancy [5].

The steroid hormone cortisol is produced by the adrenal cortex in response to stress. Cortisol’s primary functions involve affecting metabolism and the immune system, and it is involved as negative feedback in the inflammatory process [6]. Cortisol is also an important factor during pregnancy with increased levels driven by placental production of cortisol releasing factor (CRF) after the 7th week of gestation [7]. While placental CRF drives HPA output, increased placental 11 beta hydroxysteroid dehydrogenase 2 (11β-HSD2) activity protects the fetus from adverse effects of elevated systemic maternal cortisol levels [8]. Elevated maternal cortisol in early pregnancy is implicated in preterm birth as a result of an earlier than expected spike in placental CRF compared to full-term pregnancies [9]. Elevations in placental cortisol are also found in preterm birth and low birth weight pregnancies associated with preeclampsia, and suggest that cortisol may play a role in the physiological mechanism linking depression and adverse birth outcome [10].

Several factors influence the relationship between depression and birth outcome. In the US and other countries, women of low socioeconomic status (SES) have a higher rate of depression [11]. Black women have a disproportionate burden of socioeconomic stresses and poor birth outcomes [12]. It has also been shown that black women, independent of SES, report more depressive symptoms during pregnancy than white women, and are also more likely to have adverse birth outcomes [2,13]. Greater elevations in inflammatory markers and stress markers associated with depression are also more evident in black women [13]. In a longitudinal study of 2544 subjects, the inflammatory marker c-reactive protein was more closely related to depression in black compared to white subjects [13]. Obesity was also found to mediate physiologic responses to stress and that depressive symptoms are associated with increased inflammation among pregnant black women, suggesting an imbalance of pro- and anti-inflammatory factors [3]. Therefore, understanding the physiologic underpinnings linking depression, adiposity and adverse birth outcomes among black women is important in determining appropriate clinical approaches to at-risk patients during pregnancy. The focus of this study was to investigate if maternal urine cortisol in early pregnancy was significantly elevated in black women with depression compared to women without depression. In addition, we sought to compare urine cortisol values to circulating plasma values in early pregnancy, and to investigate the relationship of maternal urine and plasma cortisol values to depression and adiposity among a cohort of black women recruited in early pregnancy.

## Methods

### Subjects

The study was conducted between April 2009 and February 2013 in a sample of 50 healthy nulliparous women with a singleton pregnancy without known medical complications recruited early in the first trimester (11.2 ± 3.1 weeks gestation, average ± SD). This was a case–control study from an ongoing longitudinal investigation of pregnancy (Prenatal Exposures and Preeclampsia Prevention (PEPP)) at the University of Pittsburgh, Magee-Womens Hospital and Magee-Womens Research Institute. Subjects were recruited from the western Pennsylvania region and were all seeking prenatal care at Magee-Womens Hospital of UPMC. The ethnicity of the five county area of western Pennsylvania is primarily Caucasian (~70%) and African American (~20%). Race was self-assigned as black or African American for all subjects in this study [14,15]. Exclusion criteria included chronic hypertension, pre-gestational diabetes on medication (insulin, glyburide), major fetal anomaly or demise, planned termination of the pregnancy, collagen vascular disease (autoimmune disease) on medication, renal disease, epilepsy or other seizure disorder, active or chronic liver disease, heart disease, known illicit drug or alcohol abuse during current pregnancy. Obesity was defined as pre-pregnancy body mass index (BMI) of ≥ 30 kg/m^2^. The study was approved by the University of Pittsburgh institutional review board. Subjects were recruited at the time of presentation to Magee-Womens Hospital for prenatal care. All pregnant women were informed about the study and gave written informed consent at study enrollment.

### Assessments

All subjects were enrolled early in the first trimester, 11.3 ± 3.2 weeks gestation for the non-depressed controls and 11.1 ± 3.1 weeks gestation for depression cases. Depressive symptoms were assessed using the Edinburgh Depression Scale (EDS), a previously validated scale for detection of depression during pregnancy [16], a 10-item screening tool providing an indication of the severity of symptoms. The PEPP study was designed such that the EDS was administered to all subjects only at study enrollment. Women with EDS scores greater than or equal to 11 at the time of study enrollment were identified as depression cases (n = 25, average EDS score 14.1 ± 2.3); and this corresponded to the upper 5% of depression scores in this study population. Eight (8) of the 25 women with an EDS score of 11 or greater had a history of depression, and 1 non-depressed woman had a past history of depression treated without medication. Overall the women in this study were healthy, and none were being treated with anti-depressant medication at the time of this study. None of the women in this study were currently being treated with antidepressant medication. Subjects with depression were matched 1-to-1 by maternal age, race, pre-pregnancy body mass index (BMI), gestational age at delivery, and infant birth weight centile to non-depressed uncomplicated control pregnant women (n = 25, average EDS score 3.0 ± 2.3).

### Blood and urine samples

Maternal venous EDTA plasma samples were collected at study enrollment between 6–16 weeks gestation. In addition, a morning spot urine sample was collected at the same time as maternal EDTA plasma. Midstream clean catch urine specimens were collected first thing in the morning in the hospital clinic and processed within an hour. Samples were collected between April 2009 and February 2013 and stored at −70°C for later analysis. With the sample size of 50 women, a medium effect size of 0.30 can be detected between depressed and non-depressed women with 0.80 power at a two-tailed significance level of 0.05.

### Cortisol quantitation

Maternal plasma and urine cortisol concentrations were determined using a competitive ELISA kit from R&D Systems (KGE008, Minneapolis, MN) according to the manufacturer’s instructions. This assay has been validated. The intra- and inter-assay coefficient of variation of the assay is between 5 to 9%. The ELISA is sensitive with a reported sensitivity of 150 pg/ml compared to measured concentrations of cortisol between 14 to 100 ng/ml in plasma from pregnant women. Specificity and no cross reactivity were observed in the assay for other hormones including: prednisolone, Reichstein’s substance S, progesterone, cortisone, 4-androstene-3,17 dione, corticosterone, deoxycorticosterone, estradiol, and prednisone.

All maternal samples were analyzed in duplicate, and laboratory personnel were unaware of the case–control status of the study subjects at the time of analysis. In general, samples were diluted 1 to 20 or 1 to 40 in order for samples to fall within the linear and measurable range of the assay’s standard curve.

### Creatinine quantitation

Urine creatinine concentrations were determined using colorimetric reaction (Pointe Scientific, Canton, MI). All samples were assayed in duplicate. The average within-assay coefficient of variation for creatinine was 3.9%, and the inter-assay variability was 7.9%.

### Adiposity measures

Bioelectrical impedance analysis (BIA) was measured to determine maternal body composition and adiposity using the RJL Systems BIA Quantum IV (RJL System, Clinton Twp, MI, USA). Each participant was instructed to remove her right shoe and sock, and then lie supine with her arms away from her body. Two electrodes were placed on the right hand, one on the wrist and one on the first joint of the middle finger. Two electrodes were placed on the right foot, one on the ankle and the other on the base of the second toe. Direct measures of resistance (R), reactance (Xc), and impedance (I) were recorded. Hematocrit was measured by centrifugation of whole blood in glass capillary tubes and measured red cell volume. Fat mass and percent body fat were calculated using published formulas specific for pregnancy [17,18]. Maternal height, weight, hip circumference, and waist circumference were determined by standard clinical measures. Waist to hip ratio (WHR) was calculated using measurements conducted at study enrollment in the first trimester before the gravid uterus had begun to change the contour of the participant’s mid abdomen [19].

### Statistical analysis

Continuous data are presented as means and standard deviations (SD), and categorical data are expressed as numbers and percentages. Pregnant women with depression and non-depressed control women were matched 1-to-1 by maternal age, race, pre-pregnancy body mass index (BMI), gestational age at delivery, and infant birth weight centile and unpaired analyses were used to compare characteristics between groups (chi-square or t-test). Differences in maternal plasma and urine cortisol concentrations between women with depression and non-depressed control women were assessed using Student’s t-test or ANOVA. Correlations were by standard regression analysis with the square of the Pearson’s product moment reported (r^2^). Tests were 2-sided and statistical significance was accepted at p < 0.05.

## Results

The maternal and newborn characteristics of the study subjects are shown in Table 1. By design, depressed pregnant black women were matched to non-depressed pregnant black women by maternal age, weight, and body mass index (BMI). In addition there was no difference in maternal fat mass, percent body fat, waist/ hip ratio, blood pressure at enrollment, gestational age at delivery, infant birth weight and birth weight centile (Table 1). There was no difference in smoking status between depressed and non-depressed women. Both groups were primarily of low income, and most only had a high school education or less (Table 1). All pregnant black women in this study had uncomplicated pregnancies. Lastly, by design, the Edinburgh depression score at study enrollment was significantly elevated in the depressed pregnant black women (14.1 ± 2.3) compared to the non-depressed pregnant black women (3.0 ± 2.3, p < 0.0001).

**Table 1:** **Maternal and newborn characteristics of study subjects**

	**Non-depressed control subjects (n = 25)**	**Depressed subjects (n = 25)**
Maternal age (years)	22.2 ± 3.0	22.3 ± 3.1
Maternal weight (lbs)	179.8 ± 43.1	181.7 ± 45.4
Pre-pregnancy body mass index (BMI) (kg/m^2^)	30.3 ± 7.4	31.5 ± 7.7
Fat mass (lbs)	73.1 ± 33.3	71.4 ± 32.9
Percent body fat (%)	38.8 ± 8.1	37.4 ± 8.2
Waist/ hip ratio	0.857 ± 0.057	0.858 ± 0.080
Race (Black) n (%)	25 (100%)	25 (100%)
Average blood pressure at enrollment (mmHg)	113 ± 9/67 ± 7	110 ± 9/66 ± 7
Gestational age at delivery (weeks)	38.9 ± 1.6	39.5 ± 1.3
Infant birth weight (grams)	3254 ± 523	3274 ± 432
Infant birth weight centile (%)	58.9 ± 28.8	56.5 ± 24.4
Cigarette Smoker, n (%)	7/25 (28%)	13/25 (52%)
Household income		
<$10,000	20/25 (80%)	18/25 (72%)
$10-34,999	4/25 (16%)	5/25 (20%)
$35,000+	1/25 (4%)	2/25 (8%)
Education level		
Some high school or GED	17/25 (68%)	21/25 (84%)
Some college or associate degree	8/25 (32%)	4/25 (16%)
Edinburgh Depression Scale (EDS) score at study enrollment	3.0 ± 2.3	14.1 ± 2.3 *

Data are mean ± SD. *: *p* < 0.0001 compared to non-depressed control subjects.

### Comparison of maternal plasma and urine cortisol

**Figure 1:**
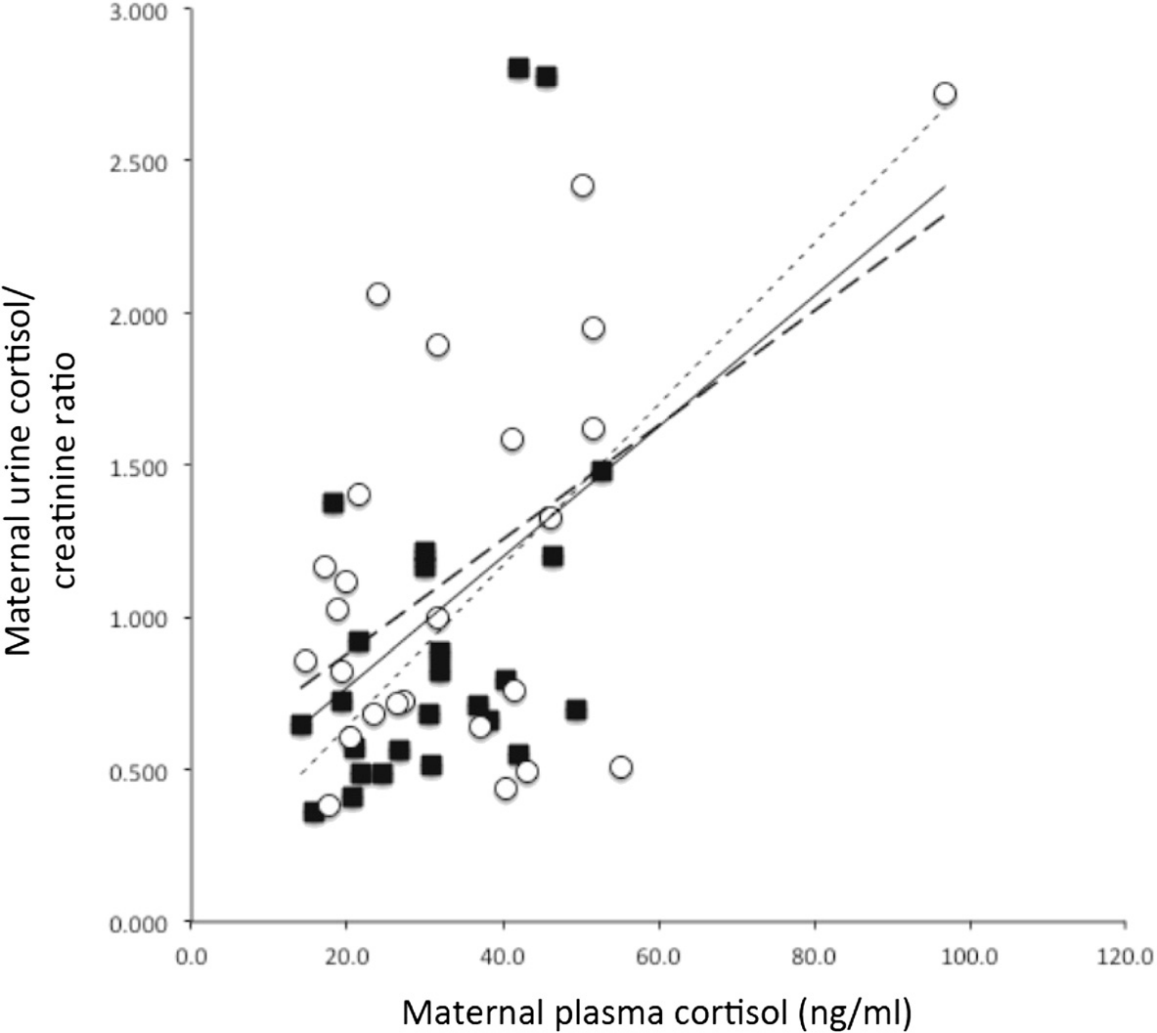
**Correlation of maternal plasma cortisol and urine cortisol between depressed and non-depressed pregnant women.** Trend line for all study subjects is the solid black line (r^2^ = 0.25, p < 0.001). Trend line for the depressed subjects only (closed symbols) is the fine dashed line (r^2^ = 0.22, p < 0.02), and the trend line for the non-depressed subjects only (open symbols) is the heavy dashed line (r^2^ = 0.28, p < 0.01).

There was no correlation between maternal plasma or urine cortisol values and gestational age at collection; plasma r^2^ = 0.03, p = 0.25 and urine r^2^ = 0.02, p = 0.29. We first set out to investigate and compare maternal urine cortisol values compared to maternal plasma samples collected at the same time. As shown in Figure 1, the maternal urine cortisol/ creatinine ratio was significantly and positively correlated to maternal plasma cortisol (r^2^ = 0.25, p < 0.001). This positive correlation between maternal plasma and the urine cortisol/ creatinine ratio was maintained in both the depressed pregnant women alone (r^2^ = 0.22, p < 0.02) and the non-depressed pregnant women alone (r^2^ = 0.28, p < 0.01).

### Maternal cortisol and depression

We next investigated differences in cortisol levels in early pregnancy between depressed and non-depressed pregnant black women. As shown in Table 2 neither plasma cortisol nor the urine cortisol/creatinine ratio was significantly different between depressed and non-depressed pregnant black women.

**Table 2:** **Differences in maternal plasma and urine cortisol measures**

	**Non-depressed control subjects (n = 25)**	**Depressed subjects (n = 25)**
Gestational age at sample collection (weeks gestation)	11.3 ± 3.2	11.1 ± 3.1
Maternal plasma cortisol (ng/ml)	31.5 (20.1, 44.7)	30.6 (21.2, 41.2)
Maternal urine cortisol/ creatinine ratio	0.99 (0.66, 1.60)	0.71 (0.55, 1.18)

Data are mean ± SD or median (interquartile range).

### Maternal cortisol and adiposity

**Figure 2:**
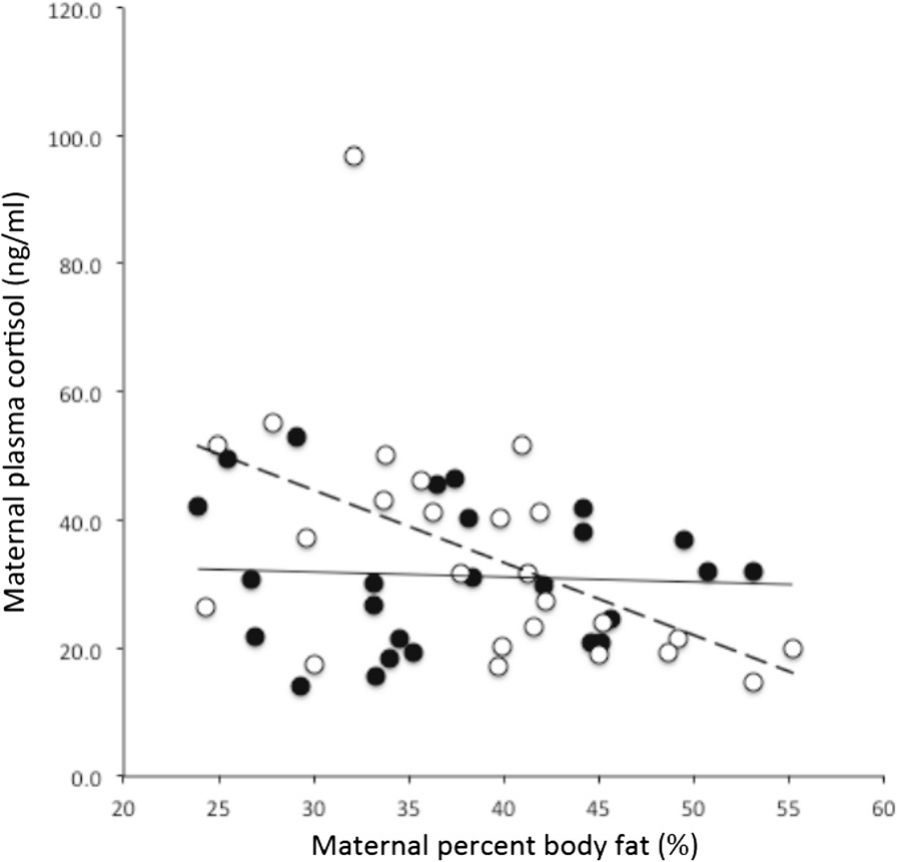
**Correlation of maternal plasma cortisol and maternal percent body fat (%) for depressed (closed symbols) and non-depressed (open symbols) pregnant women.** Trend line for the depressed subjects is the solid black line (r^2^ = 0.003, p = 0.78), and the trend line for the non-depressed subjects is the heavy dashed line (r^2^ = −0.26, p < 0.01). The correlation between plasma cortisol and percent body fat among the non-depressed women persisted after exclusion of the highest cortisol value, (r^2^ = −0.32, p < 0.01).

Several measures of adiposity were evaluated in the pregnant women in this study including BMI, waist-hip ratio (WHR), fat mass, and percent body fat. Surprisingly, despite measurements occurring in early pregnancy, there was a significant positive relationship between maternal WHR and gestational age (r^2^ = 0.11, p < 0.05), but no relationship between other measures of maternal adiposity and gestational age. There was no significant relationship between the urine cortisol/creatinine ratio and maternal measures of adiposity. In contrast to urine cortisol, maternal plasma cortisol was significantly and negatively associated with maternal weight (r^2^ = −0.10, p < 0.05), fat mass (r^2^ = −0.10, p < 0.05), percent fat mass (r^2^ = −0.10, p < 0.05), and was close to statistical significance for maternal BMI (r^2^ = −0.07, p = 0.06). Plasma cortisol was not associated with WHR (p = 0.98). Upon subgroup analysis, the relationship between plasma cortisol and maternal adiposity was only present in the non-depressed pregnant black women (weight r^2^ = −0.31, p < 0.005; BMI r^2^ = −0.29, p < 0.01, fat mass r^2^ = −0.29, p < 0.01, and percent body fat r^2^ = −0.26, p < 0.01), but not present in the depressed pregnant black women. Figure 2 illustrates the relationship between plasma cortisol and maternal percent body fat for both the depressed (p = 0.78) and non-depressed (p < 0.01) pregnant women.

### Interaction between cortisol, adiposity and depression

**Figure 3:**
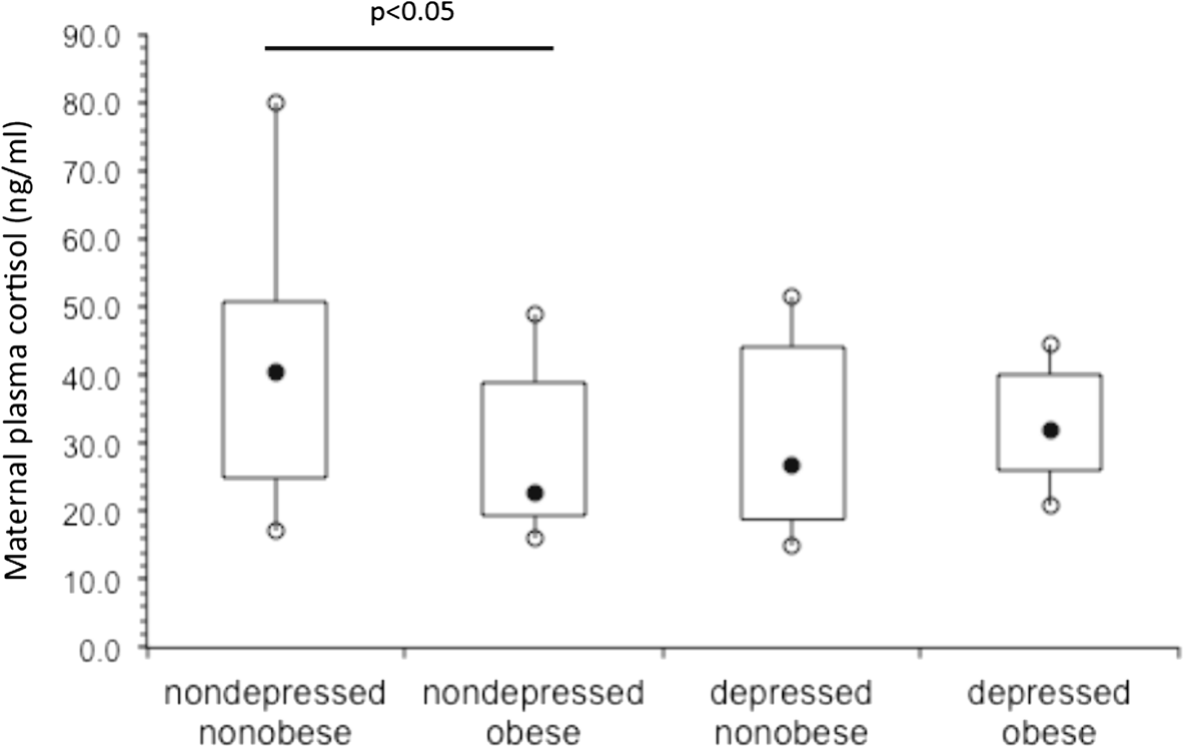
**Box and whisker plots of maternal plasma cortisol (ng/ml) by depression and adiposity.** The filled black circles are the median, the open circles are the 90th and 10th percentiles, and the top and bottom lines of the box are the 75th and 25th percentiles of the data for each group. Statistical significance is indicated by p values.

We further investigated the relationship and interaction of maternal adiposity to plasma cortisol and depression. As shown in Figure 3, maternal plasma cortisol levels were significantly elevated among the non-depressed non-obese pregnant black women (n = 13) compared to pregnant non-depressed obese women (n = 12). In contrast, there was no difference in plasma cortisol between depressed non-obese and obese pregnant black women. In addition, in a subgroup analysis, plasma cortisol was significantly elevated in the non-depressed non-obese pregnant black women compared to the other three subgroups (p < 0.05).

## Discussion

In this study we found no difference in plasma or urinary cortisol levels between depressed and non-depressed pregnant black women in early pregnancy. In contrast, plasma cortisol levels were negatively associated with several measures of maternal adiposity, and additional analysis revealed this relationship to be present only in the non-depressed pregnant black women. The HPA axis through the action of cortisol may be a mechanism by which stressors, including depression, adversely affect pregnancy and birth outcomes (e.g., preterm birth and low birth weight) [1,2,4]. This study examined the relationship and interaction between depression and maternal cortisol levels in black women in early pregnancy, including comparing early morning plasma and urine cortisol levels. These data suggest maternal cortisol values may be negatively affected by adiposity and stressors such as depression may contribute to atypical low cortisol levels.

Pregnancy is a transient hypercortisolemic state, with elevations beginning early in pregnancy, and cortisol playing a role in parturition [7]. Physiologic responses to stressors typically involve elevations in HPA axis activity and elevated cortisol. In pregnancy, placental CRF participates in a feed forward loop, and the maternal negative feedback mechanisms are down regulated leading to elevated cortisol levels [8]. Previous studies have implicated both depression and cortisol in pregnancy complications such as preeclampsia and adverse birth outcomes such as preterm birth and low birth weight [2]. Furthermore, studies have observed differences in biomarkers related to stress between depressive symptoms depending upon the study subject’s race [13]. In our study, stressors including depression and increased adiposity may blunt the physiologic HPA response in normal pregnancy and result in low morning cortisol levels.

One focus of this study was to compare maternal plasma and urine cortisol levels in order to investigate if urine cortisol measures might be an easily accessible biological material to assess maternal cortisol levels. While other studies have investigated salivary cortisol, we hypothesized maternal urine may serve as a useful depot for assessing cortisol levels. We did observe a significant positive, although modest, relationship between maternal plasma and urine cortisol levels. However, additional analyses did not provide any useful relationships between maternal urine cortisol levels and depression or adiposity among the pregnant women in this study.

The link between depression and poor birth outcomes is thought to be mediated through physiologic changes secondary to depression including elevated cortisol and pro-inflammatory factors [3]. In our study there was no significant difference in maternal cortisol levels between depressed and non-depressed pregnant black women. Previous research suggests that subtypes of depression may alter HPA axis functioning with either hyperactivity (melancholic depression) or hypoactivity (atypical depression) [20]. The depressed pregnant women in this study were assessed by the EDS, however it is possible these women may comprise a heterogenous group of depressed women including both melancholic and atypical depressive subtypes, perhaps obfuscating relationships between depression and cortisol.

While elevated cortisol is related to central adiposity in non-pregnant women [21], measures of adiposity were negatively correlated with plasma cortisol in our sample. This result may be related to the flattening of the diurnal cortisol curve observed in obese patients [22]. Typically, HPA axis dysfunction is seen in adiposity involving greater activation due to stress stimulation. In contrast, it has been found that while cortisol is positively correlated with central adiposity, the waking cortisol response can be blunted in obese subjects [22]. Our data showing a negative correlation between adiposity and morning plasma cortisol are consistent with this previous finding.

Elevated cortisol leads to central adiposity, and is associated with a flattened diurnal curve of cortisol [22]. Therefore, maternal adiposity is an expected factor contributing to atypical low morning cortisol. In addition, post-hoc analysis identified non-depressed non-obese women as having significantly elevated cortisol levels compared to depressed obese and non-obese women, and non-depressed obese pregnant black women. Depression is thought to be the result of both hyper- and hypo-responsiveness of the HPA axis [20,23]. Interestingly, while depressive subtype (atypical vs. melancholic) could be correlated with elevated or depressed HPA axis activity, depressed obese and non-obese pregnant black women in this study had lower cortisol levels when compared to non-depressed non-obese women. Atypical low morning cortisol in early pregnancy may be an indicator of HPA dysfunction, however, additional research that includes adverse pregnancy outcomes is needed to determine if these atypical cortisol levels may constitute a marker for risk later in pregnancy.

The observation of atypical morning maternal cortisol levels among pregnant depressed obese black women is unique. However, there are several limitations inherent to the current study as a result of design. A major limitation of this study is the classification of depression using the Edinburgh Depression Rating Scale. The Edinburgh scale is a screening tool for depression validated for use during pregnancy [16], but it does not replace more formal structured psychiatric assessments for depression. Of note, many of the depressed subjects in this study were noted to have a history of depression and anxiety, but this information was not complete for all subjects. An additional significant limitation of this study is the single morning urine sample which prevented us from accurately determining the diurnal variation in cortisol. In addition, the relatively small sample size of the current study is a limitation, however this study was focused solely on investigating depressed and non-depressed black women in early pregnancy which inherently limited the potential pool of subjects eligible for investigation. In addition, the current study only investigated maternal cortisol levels in uncomplicated pregnant women. However, this limitation was by design since we wished to eliminate adverse pregnancy outcomes as a potential confounder of dysregulated cortisol levels in pregnancy. Similarly, this study only investigated pregnant black women; however again this limitation was by design since we were specifically interested in and hypothesized that atypical and dysregulated maternal cortisol levels would be evident among pregnant black women. Lastly, while all urine samples in this study were early morning collections, we were unable to obtain samples collected by standardized methods. Despite this limitation we did observe a significant and positive correlation between urine and circulating plasma cortisol levels.

## Conclusions

In this case–control study we investigated cortisol levels in pregnant women with uncomplicated pregnancies to examine the relationship between adiposity, depression and cortisol. We observed elevated circulating maternal cortisol levels in non-depressed non-obese pregnant black women compared to depressed obese pregnant black women, consistent with elevated cortisol noted in pregnancy. In addition, we observed an atypical blunting of maternal cortisol levels among obese depressed and non-depressed pregnant black women and non-obese depressed pregnant black women. We hypothesize these data indicate HPA axis dysfunction in early pregnancy. Both depression and adiposity may influence this relationship. Future studies should further investigate this relationship and should include women with adverse pregnancy outcomes including preterm birth and fetal growth restriction.

## Abbreviations

11β-HSD2: 11 beta hydroxysteroid dehydrogenase 2
BIA: Bioelectrical impedance analysis
BMI: Body mass index
CRF: Cortisol releasing factor
EDS: Edinburgh Depression Scale
HPA: Hypothalamic-pituitary axis
PEPP: Prenatal Exposures and Preeclampsia Prevention
SES: Socioeconomic status
SD: Standard deviation
WHR: Waist-hip ratio
